# Long-term treatment of dentine with triple antibiotic paste promotes stem cell viability and attachment

**DOI:** 10.1016/j.jtumed.2022.01.007

**Published:** 2022-02-10

**Authors:** Samiya Riaz, Ahmad Azlina, Zuliani Mahmood, Aung T. Htun

**Affiliations:** a Paediatric Dentistry Unit, School of Dental Sciences, Universiti Sains Malaysia, Health Campus Kubang Kerian, Kelantan, Malaysia; b Dental Clinics, Hospital Universiti Sains Malaysia, Health Campus Kubang Kerian, Kelantan, Malaysia; c Basic Sciences and Oral Biology Unit, School of Dental Sciences, Universiti Sains Malaysia, Health Campus Kubang Kerian, Kelantan, Malaysia; d Human Genome Centre, School of Medical Sciences, Universiti Sains Malaysia, Health Campus Kubang Kerian, Kelantan, Malaysia

**Keywords:** Cell viability, Dental pulp stem cells, Intracanal medicaments, Irrigated radicular dentine, Scanning electron microscope, قابلية حياة الخلية, الخلايا الجذعية للب الأسنان, الأدوية داخل القناة, عاج جذري مروي, المجهر الإلكتروني الماسح

## Abstract

**Objective:**

Successful regenerative endodontic procedures in dental treatment are critically associated with complete disinfection of the root canal and require irrigants and medicaments. One factor for consideration is the biocompatibility of the medicament as this can affect the intracanal dentine and subsequently the dental stem cell viability required for the repair of the dentine–pulp complex. This in vitro study investigated the effect of a 4-week treatment of calcium hydroxide [Ca(OH)_2_] and triple antibiotic paste (TAP) on the irrigated radicular dentine by analysing dentine interaction with dental stem cells.

**Methods:**

TAP consists of metronidazole, ciprofloxacin and minocycline. Dentine chips were prepared and treated with either Ca(OH)_2_ or TAP for 4-weeks, irrigated by 1.5% sodium hypochlorite (NaOCl), rinsed with saline, followed by 17% ethylenediaminetetraacetic acid (EDTA). Dental pulp stem cells (DPSCs) cultured on the surface of the dentine chips were analysed on days 1, 3 and 7 of cell seeding for PrestoBlue viability assays, 6-diamidino-2 phenylindole (DAPI) staining and scanning electron microscopy (SEM). An independent t-test (SPSS software version 24.0) was used to statistically analyse the PrestoBlue assay data.

**Results:**

DPSCs grown from dentine treated with TAP showed significantly higher cell viability than the Ca(OH)_2_ and control groups (*p* < 0.05). DAPI staining of the seeded DPSCs on the treated dental chips complemented the findings of the viability assay. SEM studies also revealed improvements in the cell spreading and attachment of DPSCs grown on TAP-treated dentine compared with Ca(OH)_2_.

**Conclusion:**

The treatment of dentine with TAP for 4 weeks provided a better microenvironment for the viability and attachment of DPSCs when compared to Ca(OH)_2_.

## الملخص

**أهداف البحث:**

ترتبط إجراءات اللبية التجديدية الناجحة في علاج الأسنان ارتباطًا وثيقًا بالتطهير الكامل لقناة الجذر، الأمر الذي يتطلب الري والأدوية. أحد العوامل التي يجب مراعاتها هو التوافق الحيوي للدواء، والذي يؤثر على العاج داخل القناة وبالتالي على قابلية الحياة للخلايا الجذعية السنية اللازمة لإصلاح مجمع لب العاج. بحثت هذه الدراسة في المختبر تأثير العلاج لمدة 4 أسابيع لهيدروكسيد الكالسيوم ومعجون المضاد الحيوي الثلاثي، على العاج الجذري المروي عن طريق تحليل تفاعل العاج مع الخلايا الجذعية السنية.

**طرق البحث:**

يتكون المعجون الثلاثي من ميترونيدازول وسيبروفلوكساسين ومينوسكلين. تم تحضير رقائق العاج ومعالجتها باستخدام هيدروكسيد الكالسيوم أو المعجون الثلاثي لمدة 4 أسابيع، مروية بنسبة 1.5٪ هيبوكلوريت الصوديوم، وشطفها بمحلول ملحي، ثم بحمض إيثيلين ديامينيتتراسيتيك (تركيز 17٪). تم تحليل الخلايا الجذعية للب الأسنان المزروعة على سطح رقائق العاج في اليوم 1 و 3 و 7 من بذر الخلايا لفحص قابلية الحياة (بريستوبلو)، وصبغة فينيل إندول، والمسح المجهري الإلكتروني. تم استخدام اختبار تي المستقل لتحليل بيانات اختبار بريستوبلو إحصائيا.

**النتائج:**

أظهرت الخلايا الجذعية للب الأسنان المزروعة من العاج المعالج بـالمعجون الثلاثي قابلية حياة أعلى بكثير من مجموعة هيدروكسيد الكالسيوم ومجموعات التحكم. وأكدت نتائج صبغة فينيل الإندول للخلايا الجذعية للب الأسنان هذه النتائج. كما كشفت دراسات المسح المجهري الإلكتروني أيضا عن انتشار أفضل للخلايا المعالجة بـالمعجون الثلاثي مقارنة بالمجموعة المعالجة بهيدروكسيد الكالسيوم.

**الاستنتاجات:**

عاج الأسنان المعالج بـالمعجون الثلاثي لمدة 4 أسابيع يوفر بيئة أفضل لقابلية الحياة للخلايا المعالجة بـالمعجون الثلاثي مقارنة بهيدروكسيد الكالسيوم.

## Introduction

Pulp inflammation and necrosis, resulting from infection in immature permanent teeth with open apices, usually lead to inhibition of root maturation. As a consequence, the tooth is prone to fractures due to the thin dentinal walls. Regenerative endodontic procedure (REP) is a relatively new clinical procedure that makes use of tissue engineering technologies. The goals of RE are to eliminate infective clinical symptoms, heal the dentine–pulp complex and the periapical tissues, and promote continued root maturation and development.^1^

One of the main focuses in REPs is canal disinfection. Therefore, REPs are particularly relevant to two critical steps during canal preparation: irrigation and placement of intracanal medicaments. The use of suitable irrigants improves the effects of intracanal medicaments. The function of these irrigants is to remove the smear layer and expose the dentinal tubules, thus promoting the attachment of the stem cells to the dentine.^2^^,^^3^ Intracanal medicament eliminates bacteria and provides the canal system with an optimal environment for the growth of stem cells. The selection of irrigants and the period for treatment with intracanal medicaments to function effectively should be based on their bactericidal/bacteriostatic properties and their ability to promote the survival and proliferative capacity of the stem cells. The absorption of the irrigants into dentinal root surfaces at certain concentrations, even when the agents are not admixed, combined with the duration for intracanal medicament treatment, however, may cause chemical interactions, forming unwanted by-products that may be toxic or irritant to the stem cells.^4^ Since REPs are stem cell-based therapies, the full potential of these procedures is dependent on the ideal interaction of the bioengineering triad: cells, scaffold and growth factors.^1^ Thus, for REPs, the stem cells must survive disinfection protocols in order to participate in tissue regeneration. Therefore, in this study, we investigated the durability of dental stem cells.

Calcium hydroxide [Ca(OH)_2_] and triple antibiotic paste (TAP) (consisting of metronidazole, ciprofloxacin and minocycline) have been widely used as intracanal medicaments. The American Association of Endodontists (AAE) recommends the application of intracanal medicament for at least 1 week and a maximum of 4 weeks.^5^ The observed variation in clinical outcomes created a critical gap regarding the effects of these medicaments on stem cell survival and attachment to the root canal of the irrigated dentine.^6^^,^^7^ Some lines of evidence support the use of Ca(OH)_2_,^8^, ^9^, ^10^, ^11^^,^ although a few studies have also indicated that TAP enhanced the attachment of dental pulp stem cells (DPSCs) to the dentine.^12^ These authors also found that double antibiotic paste (DAP) and Ca(OH)_2_ reduced the proliferation of DPSCs.^12^ However, these findings were controversial due to the different experimental protocols used and the evaluation of stem cell survival after the short-term application of intracanal medicaments over 3- and 7-day periods.^11^, ^12^, ^13^

Currently, information relating to the effectiveness of the entire endodontic regeneration protocol is scarce.^13^^,^^14^ While more in vivo studies and clinical research are needed, the results arising from in vivo and in vitro experiments showed that multiple irrigants and medicaments exert biological effects on human DPSCs and other stem cells relating to their migration, proliferation and differentiation.^14^ Due to limitations associated with the conduction of clinical studies, we aimed to address this issue by using an in vivo assay to revaluate the disinfection protocol in a controlled microenvironment. Therefore, this study aimed to compare a 4-week (i.e., the maximum period recommended by the AAE) treatment effect of Ca(OH)_2_ and TAP on the dentine tooth surface and the viability and attachment of DPSCs. Our overall goal was to develop an optimum treatment protocol that enhances the survival of stem cells during REPs.

## Materials and Methods

### Materials

We used extracted human teeth in this study. We collected sound premolar teeth that were extracted in an atraumatic manner for orthodontic treatment from patients who were 16–30 years of age. This age limitation reduced the differences between young and aged dentine structures in their microscopic and microstructural contents since the collection of immature permanent teeth was not feasible. Teeth were excluded if they presented with carious lesions or any other pulpal pathology, or if they were cracked or fractured, periodontally compromised, or if there was the presence of restorations or treated roots or double rooted teeth with bifurcations extending beyond the apical portion towards the middle and coronal portion. After obtaining informed consent from the patients, 30 extracted sound human premolars were collected from the Family Medicine Clinic of Hospital Universiti Sains Malaysia. The teeth were stored in 2.5% NaOCl solution for 24 h for disinfection and the elimination of contamination. Subsequently, the teeth were transferred to sterile water at 4 °C till further use.

### Dentine chip preparation

**Figure 1:**
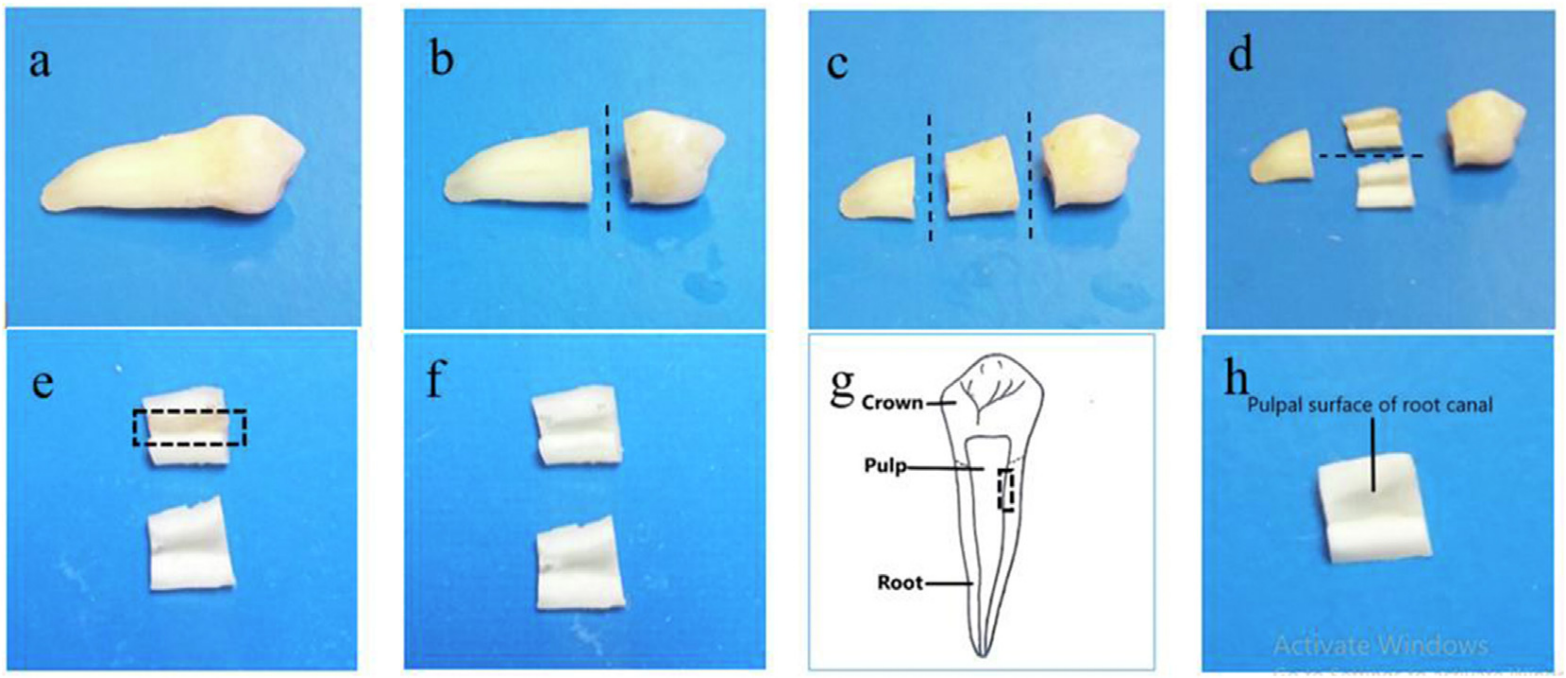
The steps taken to prepare dentine chips. (a) Sound premolar. (b) Resection of the coronal third. (c) Resection of the apical third. (d) Vertical sectioning of the middle third. (e) Middle resected portions with dental pulp; the box of dotted lines represents the dental pulp in the root canal. (f) Pulp removed from the middle portion. (g) The area from which the dentine chip was prepared. (h) Reduction of the middle resected portion to a standardized dentine chip (4 × 4 mm).

Soft gingival tissue, periodontal ligament and debris were removed from the tooth. The dentine samples were then prepared using a water-cooled hard tissue cutter (Exact, Germany). First, the tooth was horizontally sectioned into three parts: coronal, middle and apical third [Figure 1(b, c)]. Next, the sections of teeth were sliced vertically into two sections [Figure 1(d)]. Pulp from the root canals of the prepared dentine sample was gently removed using a sterile tweezer [Figure 1(e, f)]. Finally, the samples were standardised into a 4 × 4 mm sample [Figure 1(g, h)]. The apical portion was not used since it could have an open or closed apex and therefore could not be standardised. We used the middle part as this gave us a standard size and area for the placement of medicaments and the attachment of stem cells. The dentine chips were then stored independently in sterile water at 4 °C until further use.

### Irrigation, medicament preparation and the treatment of the dentine chips

Dentine specimens were first subjected to irrigation. For irrigation, the prepared dentine chip samples were immersed in 10 ml of 1.5% sodium hypochlorite solution (NaOCl) for 5 min, followed by immersion in 10 ml of normal saline (NS) for 5 min. After irrigation, the samples were randomly divided into three groups; irrigation only (I.O.), irrigation and Ca(OH)_2_ treatment (I.C.), and irrigation and TAP treatment (I.T.); cell only (C.O.) was used as a control group. Then, we set up the respective intracanal medication treatments.

Two types of intracanal medicaments were used in this study. The first intracanal medicament was a commercial form of Ca(OH)_2_ (UltraCal™ Xs, USA), with a concentration of Ca(OH)_2_ of 1 g/ml and a pH of 12.5 and is available in the form of a paste loaded in a syringe. This material was pipetted directly onto the dentine chips. The second intracanal medicament used was TAP. This was prepared using a combination of three antibiotic constituents, i.e., metronidazole tablets (200 mg) (Intas Pharmaceuticals Ltd, Malaysia), minocycline capsules (100 mg) (Pharma Health, Pakistan), and ciprofloxacin tablets (500 mg) (Pharmaniaga, Malaysia), using a separate mortar and pestle for each. Then, 100 mg of each powder was combined in a container to obtain 300 mg of antibiotic powder. This powder was then dissolved in 300 ml of distilled water to yield 1 mg/ml of TAP (with a watery consistency). We selected a concentration of 1 mg/ml for this study as this is the lowest concentration of TAP recommended by the AAE guidelines.^5^

Irrigated dentine chips were placed individually in a separate well on a 24-well plate. For the control (I.O.) group, the irrigated dentine samples were soaked in 1 ml of normal saline. For the I.C. group, 200 μl of Ca(OH)_2_ paste was applied onto each irrigated dentine chip. For the I.T. group, each irrigated dentine specimen was immersed in 1 ml of TAP. All dentine chip samples were then placed in an incubator at 37 °C in a humidified atmosphere of 5% CO_2_ for 4 weeks, denoting the period of intracanal medicament treatment.

After 4 weeks of incubation for the medication treatment, dentine chip specimens were transferred into new well plates and irrigated with 10 ml of normal saline for 5 min. Next, all dentine chip samples were irrigated with 10 ml of 17% EDTA for 5 min. To confirm the complete removal of medicaments from the dentine chips, the medicament layer was gently peeled off from the treated surface of the dentine chip prior to EDTA irrigation. After 5 min, the dentine chips were removed from the well plates and air-dried on a filter paper. The samples were then stored in a sterile container at 4 °C until further use.

### Cell culture

Commercial DPSCs (AllCells, USA) were used from passage 7 to 9 at 70%–80% confluency. DPSCs were cultured in a complete medium consisting of alpha-minimum essential medium (α-MEM) (a modification of minimum essential medium) supplemented with 10% fetal bovine serum (FBS) and 1% Pen/Strep incubated in a 37 °C humidified incubator with 5% CO_2_ in T-25 flasks. The medium was changed every third day until the cells reached 70%–80% confluency.

### PrestoBlue cell viability assay

In order to determine the viability of DPSCs cultured on dentine chips with different treatments, we conducted the PrestoBlue Cell Viability assay (Cell Viability assay, Invitrogen) on days 1, 3 and 7 of cell seeding, respectively. We freshly prepared a master mix solution in which 10% of PrestoBlue reagent was added with 90% of culture medium. Then 1 ml of the master mix solution was transferred to a 24-well plate and incubated for 2 h in a humidified incubator at 37 °C with 5% CO_2_. The assay was run in triplicate for all groups. After 2 h, 100 μl of the mixture was transferred to a 96-well plate (eight replicates). Next, the 96-well plate containing the mixture was read using an ELISA microplate reader at a wavelength of 570 nm; 600 nm was used as a reference wavelength. The absorbance of blank media was subtracted from sample values to normalise the data. The values were calculated and indexed using the following formula:

$$\text{Index}\;\text{value}=\frac{\text{Absorbance}\;\text{of}\;\text{treated}\;\text{well}-\text{blank}\;\text{well}}{\text{Absorbance}\;\text{of}\;\text{control}\;\text{well}-\text{blank}\;\text{well}}$$

### Nucleus fluorescence staining using DAPI

Dentine chip samples from each group were seeded with DPSCs from passage 7 to 9 at a concentration of 1 × 10^4^ and harvested on day 3. The samples were washed thrice with PBS in a 24-well plate and fixed with 3.7% formaldehyde for 10 min at room temperature. The samples were then washed twice with phosphate-buffered saline (PBS). Finally, each sample was placed on a separate glass slide, and two drops of mounting medium Fluoroshield™ with 6-diamidino-2 phenylindole (DAPI) (Sigma-Aldrich, Germany) was added onto each sample to stain and mount them. The samples were then covered with a coverslip, sealed using nail polish and kept in darkness for 20 min. Finally, the samples were observed under an Axioplan fluorescence microscope (Zeiss, Germany).

### Scanning electron microscopy for the assessment of cell attachment

Scanning electron microscopy (SEM) was conducted to examine the dentine surface of the experimental teeth. After cell seeding, samples from each group were harvested on days 1, 3 and 7. A dentine sample without cells was prepared as a control. First, the samples were washed three times in PBS. Then, the samples were fixed in 4% paraformaldehyde in 0.1 M phosphate buffer for 2 h at room temperature. They were then rinsed with PBS, followed by incubation in 8% formaldehyde for 2 days at 4 °C. This was followed by dehydration in a series of ethanol solutions (30%, 40%, 50%, 70%, 90%, 100%), each for 10 min. Next, the samples were incubated in hexamethyldisilane for 10 min, followed by 10 min in a desiccator. Dentine chips were then coated with a 20–30-nm thin metallic layer of gold in a sputter coating machine (Leica EM SCD, Czech Republic), and scanning was carried out using Quanta FEC 450. SEM micrographs were captured at 1000× and 2500× magnifications. Each dentine chip specimen was scanned in its entirety to obtain an overview of the general surface topography.

### Statistical analysis

All experiments were performed in triplicate (final n = 9) and the mean values were calculated. The viability of the DPSCs was analysed using an independent t-test with a significance level of 5%.

## Results

### Cell viability analysis of DPSCs cultured on treated dentine chips

**Figure 2:**
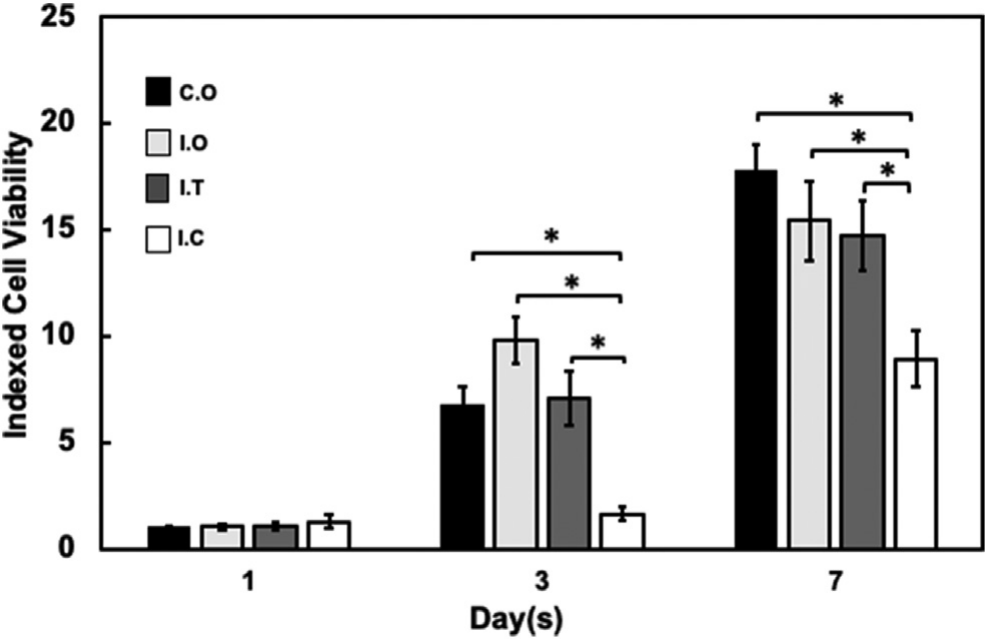
Cell viability assay of dental pulp stem cells (DPSCs) cultured on treated irrigated dentine chips. The PrestoBlue assay was performed on the cells on days 1, 3 and 7 from cell seeding. Data are presented as the mean ± standard error of the mean of the optical density at the absorbance of 570 nm wavelength normalized to a wavelength of 600 nm ∗Significant difference (*p* < 0.05) between groups. C.O., cells only; I.O., cells seeded on irrigated dentine chips; I.T., cells seeded on an irrigated dentine chip and treated with triple antibiotic paste; I.C., cells seeded on an irrigated dentine chip and treated with Ca(OH)_2_.

In vitro cell viability analysis of DPSCs was conducted to evaluate the cytotoxic effects of the intracanal medicaments on the cells; for this, we assumed that the medicament might be absorbed into the dentine. The viability of cells is known to be reduced if the cells experience a cytotoxicity effect. The number of viable DPSCs on the dentine chip samples was evaluated using the PrestoBlue viability assay at different time points (days 1, 3 and 7). All results were calculated as index values and divided by the positive control values. Analysis is shown in Figure 2. On day 1, there was no significant difference between any of the groups. However, on day 3 and day 7, the I.C. group showed significantly lower cell viability than the other groups (p < 0.05). The number of DPSCs in the group treated with TAP on day 1 showed a significant increase when compared with day 3 (p < 0.05), but was similar to the control and I.O. groups. However, there was no significant increase in cell viability in the I.C. group when compared between day 1 and day 3. Although there was a significant increase in the number of DPSCs between day 1 and day 7 (p < 0.05) and between day 3 and day 7 (p < 0.05) in the I.C. group, it was significantly lower than the I.T. group on day 3 (p < 0.05) and day 7 (p < 0.05). This finding indicated that the cell viability of DPSCs cultured on dentine chip specimens treated with TAP was higher than those treated with Ca(OH)_2_.

**Figure 3:**
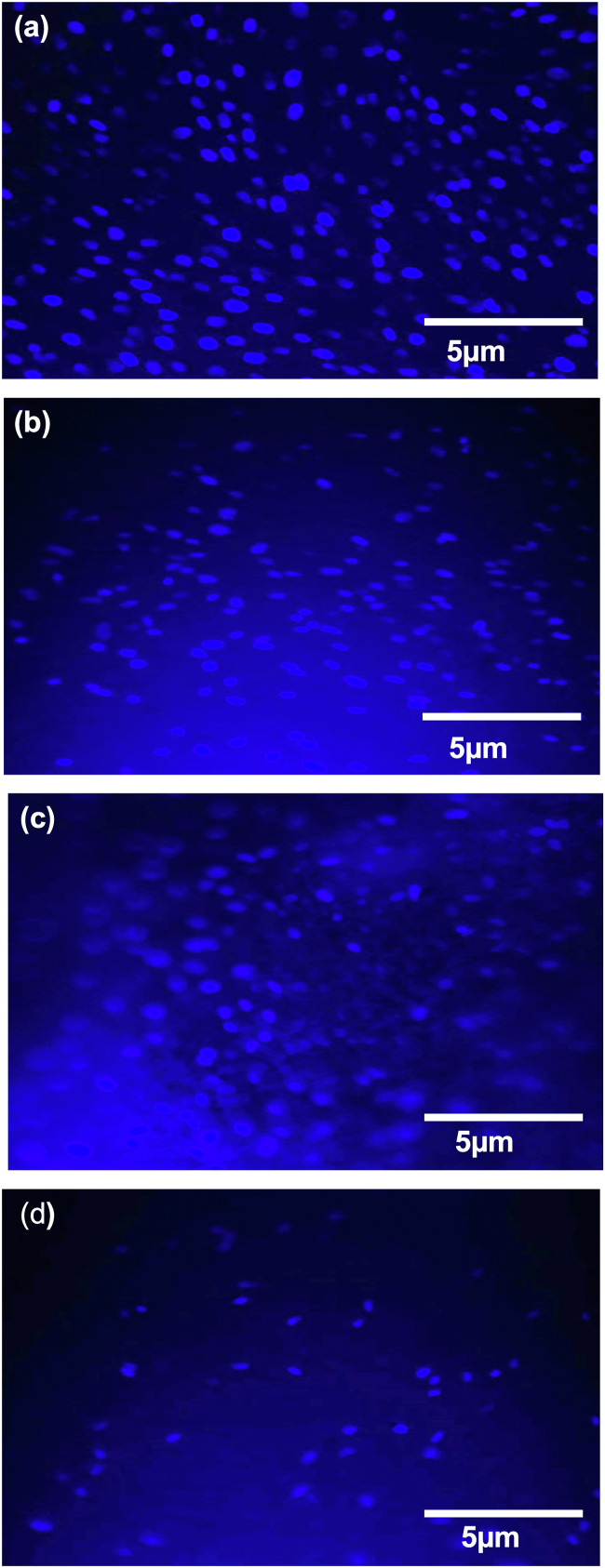
Images of stained DPSCs that had been cultured on treated irrigated dentine chips using 6- diamidino-2 phenylindole (DAPI). (a) C.O., cells only; (b) I.O., cells seeded on irrigated dentine chips; (c) I.T., cells seeded on an irrigated dentine chip and treated with triple antibiotic paste; (d) I.C., cells seeded on an irrigated dentine chip and treated with Ca(OH)_2_. Scale bar: 5 μm. Magnification 100×.

DAPI nucleus fluorescence staining was conducted to identify the viable cells grown on the dentine chips in a quantitative manner. Based on viability assay results, the samples of DPSCs samples were stained on day 3 only because stained nuclei can be differentiated (Figure 3). The control group was cells grown on glass covers (C.O.). The presence of a light blue nucleus, as seen under a fluorescence microscope, indicated the presence of viable cells (Figure 3a). The I.T. group showed the highest number of prominent light blue nuclei distributed on the slide than any of the other groups (Figure 3b). Very few stained nuclei were seen in the I.C. group when compared with the other groups (Figure 3d). Based on this observation, the rank order of the groups from the highest to the lowest number of stained nuclei was C.O., I.T., I.O. and I.C.

### Cell attachment

**Figure 4:**
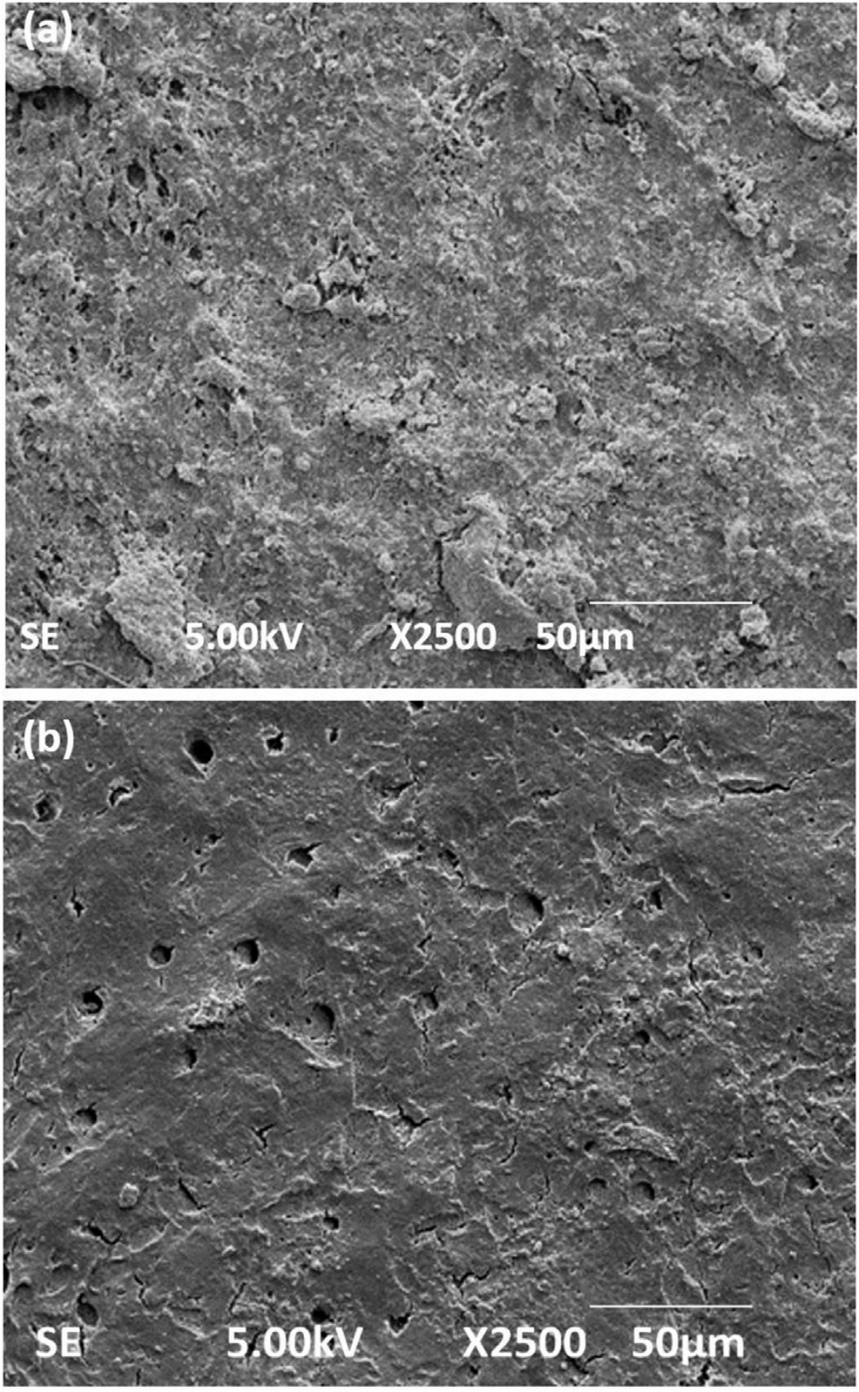
Scanning electron microscope images of the dentine surface before and after irrigation. (a) The image showing a dentinal surface completely covered with a smear layer blocking all of the dentinal tubules before smear removal, magnification 2500×, (b) After irrigation, the exposed dentinal tubules were seen due to smear layer removal in some of the areas, magnification 2500×, scale bar; 50 μm.

Figure 4a shows the surface of a dentine chip specimen before irrigation, where it was completely covered by the smear layer blocking the dentinal tubule openings. After irrigation, the smear layer was partially removed from the dentine surface (Figure 4b) and had exposed dentinal tubules with intact circular dentinal openings. On day 1, the cultured cells were seen attached to the dentine surface regardless of the presence or absence of the smear layer.

**Figure 5:**
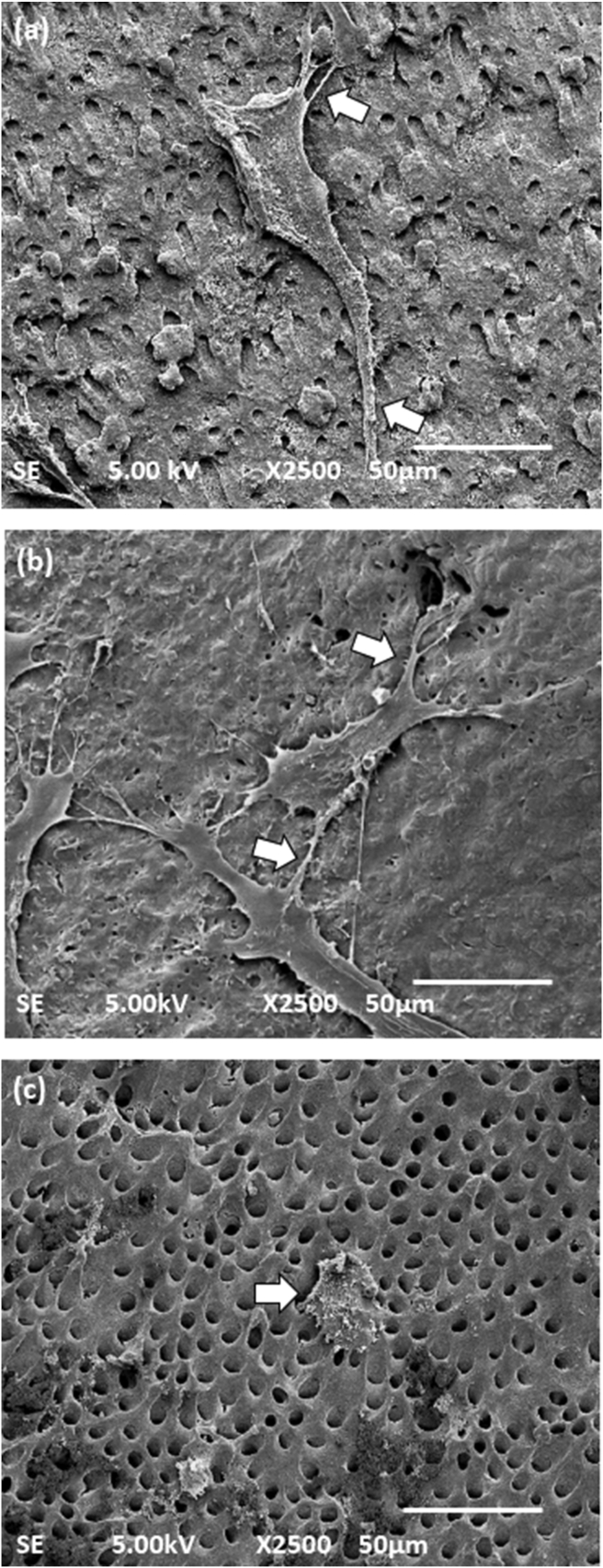
Scanning electron microscope images of dental pulp stem cells seeded on irrigated dentine chips with or without treatment on day 1. (a) The I.O. group had round cells to almost well spread cells attached to the dentinal surface with lamellipodia (white arrows). (b) In the I.T. group, the cells had already flattened in most areas. The cells were attached to the dentinal surface with lamellipodia (white arrows) and filopodia. Dentinal tubules were exposed in some areas. (c) In the I.C. group, few cells were observed on the dentinal surfaces with round shaped morphology (white arrows). The smear layer had been partially removed. Magnification, 2500×, scale bar; 50 μm.

On day 1 (Figure 5), more cells were observed in the I.O. group (Figure 5a) and the I.T. group (Figure 5b) in comparison with the I.C. group (Figure 5c). Observation of both the I.O. and I.T. groups showed the cell attachment on the dentine surface with filopodia extending into the dentinal tubules. Most cells were rectangular with unipolar lamellipodia, which were evident as thin membranous protrusions (white arrows). However, on I.C.-treated dentine, many round shaped non-spreading cells were still observed (white arrows), with many openings of the dentinal tubules. We observed that most of the dentinal tubule openings shown in Figure 5b were covered with a smear layer.

**Figure 6:**
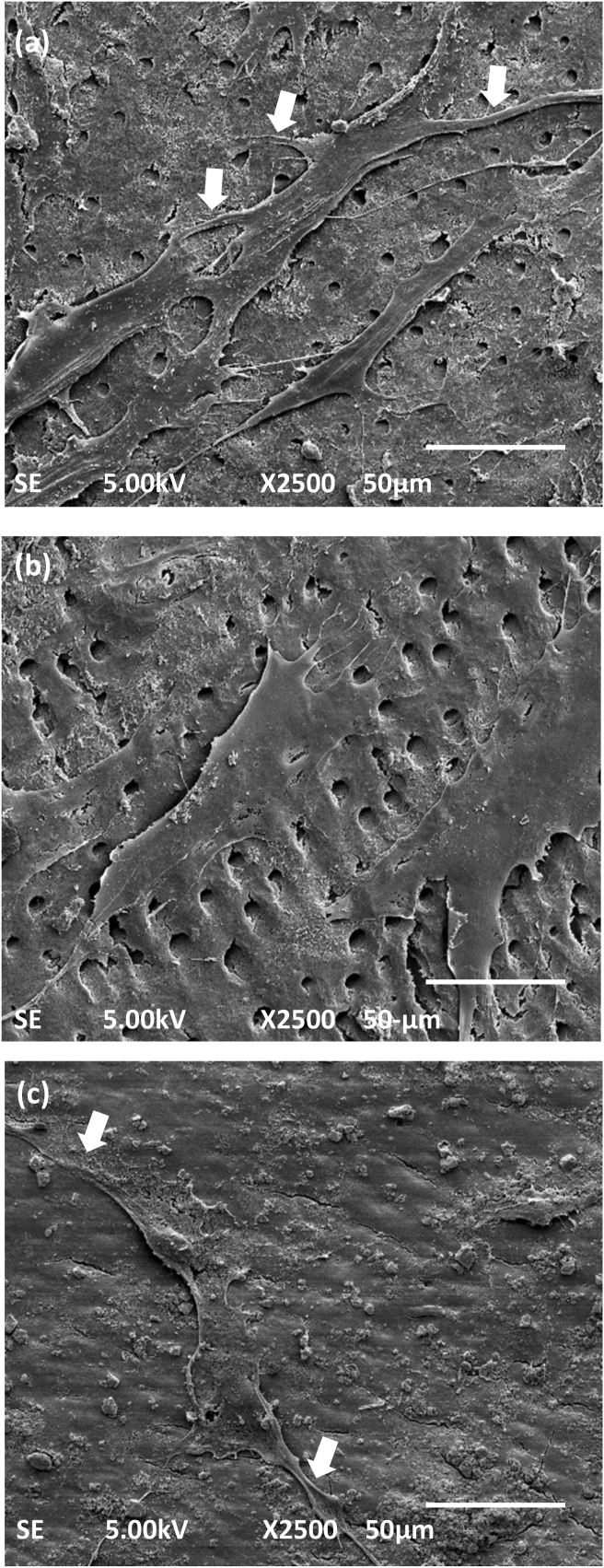
Scanning electron microscope images of dental pulp stem cells seeded on irrigated dentine chips with or without treatment on day 3. (a) In I.O. groups, the cells were almost well spread and had acquired fibroblast-like morphology with lamellipodia (white arrows) and filopodia-like structures. (b) The I.T. group showed many well spread cells with multiple lamellipodia. (c) In the I.C. group, very few rectangular cells were attached to the dentinal surface with their lamellipodia (white arrows). Magnification, 2500×, scale bar; 50 μm.

DPSCs on day 3 in the I.O. group (Figure 6a) were shown to be well spread with numerous lamellipodia extensions (white arrows) and filopodia-like structures (white arrows) progressing into the exposed open dentinal tubules of the dentine surfaces. We observed that some cells were connected with the extension of filopodia. However, the DPSCs in the I.T. group (Figure 6b) were better spread than the I.O. group because the cell margin was not well defined. Nevertheless, the I.C. group showed few rectangular cells with very few lamellipodia projections (Figure 6c) on the dentine surfaces. Most of the dentinal tubule openings shown in Figure 6c were also covered with a smear layer.

**Figure 7:**
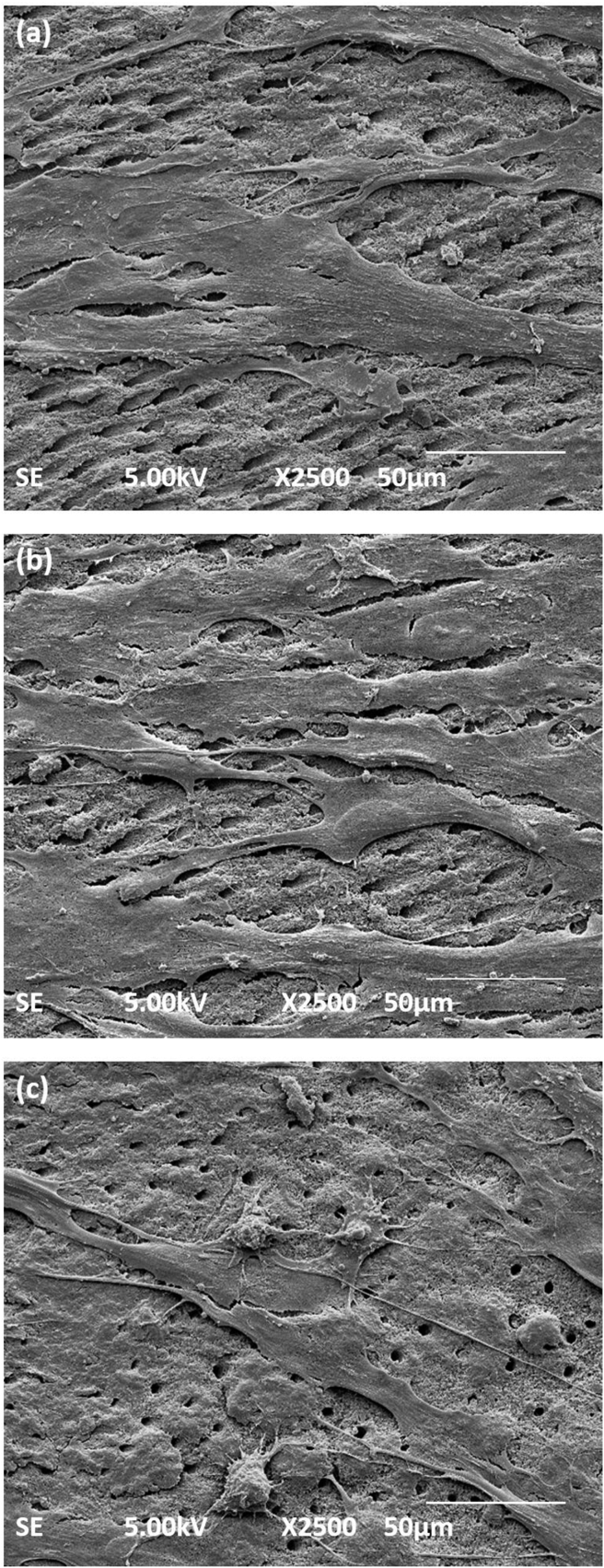
Scanning electron microscope images of dental pulp stem cells seeded on irrigated dentine chips with or without treatment on day 7. (a) Dentine in the I.O. group had flat well-spread and overlapping cells in some areas with multiple lamellipodia. (b) The I.T. group had fibroblast-like well-spread cells that overlapped in most of areas with indistinguishable margins. (c) The I.C. group had round-shaped cells while some cells were well spread with distinguishable margins. Magnification, 2500×, scale bar; 50 μm.

Observation on day 7 showed that DPSCs in the I.O. group were well spread with overlapping cells, while some of the cells still showed identifiable cell margins (Figure 7a). The I.T. group showed overlapping of well-spread flattened spindle-shaped cells on the dentine chips in most of areas (Figure 7b). In addition, we were not able to distinguish their margins. By contrast, some of the cells in the I.C. group still showed round-shaped features, while others were well spread. The margins of the cells were still distinguishable in most of the areas (Figure 7c). In general, scanning electron microscope images suggested that the attachment of DPSCs on dentine specimens treated with I.T. was better than that in the I.O.- and I.C-treated groups. Figure 7c also shows that the surface was covered mainly by the smear layer.

## Discussion

In the current study, we investigated the effect of medicaments on the dentine, as evidenced by the viability and attachment of DPSCs on the treated dentine. The usage of dentine mimics the in vivo environment while interacting with DPSCs.

Irrigation is the process used to remove a smear layer from the dentine surface which allows tubules to open to permit promoting factors to induce the growth of DPSCs. Thus, the use of biocompatible irrigants is essential for REPs.^15^ NaOCl works well as an irrigant but has a poor capability to dissolve the inorganic contents present in the smear layer.^2^ Thus, EDTA is used to facilitate the removal of the inorganic components.^16^ However, although 17% EDTA was used in this study, it did not result in complete removal of the smear layer. Similar to other studies, some parts of the smear layer still blocked the dentinal tubule openings.^17^^,^^18^ Nevertheless, the presence of a smear layer does not have a significant effect on cell attachment.^17^ Thus, the presence of a smear layer and cell attachment was not investigated in the current study.

NaOCl is toxic to cells as an irrigant,^4^^,^^19^^,^^20^ despite being an excellent antimicrobial agent^21^ and commonly used in REPs.^22^ Previous research showed that a high concentration of NaOCl is toxic to dental pulp stem cells and affects the odontogenic differentiation of cells.^23^ NaOCl was used at a concentration of 1.5% in this study because this concentration is considered significant for disinfection while maintaining its biocompatibility.^20^^,^^24^ Therefore, the second EDTA step could also serve to remove remnants of NaOCl.^20^^,^^25^

In this study, the attachment and viability of DPSCs was significantly improved when cultured on a TAP-treated dentine sample. These findings suggest that TAP is a better medicament when compared with Ca(OH)_2_. Biochemically, the low pH of TAP causes acidic demineralisation of the irrigated dentine.^26^ Demineralisation may release growth factors such as transforming growth factor (TGF)-β that become entrapped in the hydroxyapatite of the dentine^27^ during dentinogenesis.^28^^,^^29^ The release of the significant remnants of such growth factors might induce DPSCs for the differentiation of odontoblast-like cells.^26^^,^^27^ Moreover, the demineralisation of the dentine surface may increase surface roughness which is suitable for cell attachment.^30^^,^^31^

From the chemical point of view, Ca(OH)_2_ is a slow-acting chemical that requires time to degrade the dentinal proteins and reduce the mechanical properties of dentine.^32^ The alkaline pH of Ca(OH)_2_ causes denaturation of collagen proteins,^27^ which may disrupt the mineralised dentine by increasing the elastic modulus of the dentine^33^ and decrease the wettability of the dentine surface.^31^ Hence, the mechanical environment becomes unsuitable for cellular attachment and proliferation. Furthermore, denaturation of the collagen proteins by Ca(OH)_2_ may have led to the loss of substantial remnants of the growth factors, thus making them unavailable for cell growth and attachment.^13^ Since demineralisation is essential to promote the release of the growth factors and other signalling molecules,^34^ the growth of cells is affected in a negative manner, as demonstrated in the current study.

Previous studies have highlighted the excellent antibacterial properties of TAP^26^^,^^35^; disinfection with TAP might provide a preferable microenvironment that is suitable for the growth and proliferation of DPSCs. Thus, TAP had a more favourable effect on the viability and attachment of DPSCs in comparison with Ca(OH)_2_; these findings concur with those reported in several other studies.^12^ In the current study, the pattern of attachment and the morphology of the attached cells was different on TAP-treated dentine when compared with Ca(OH)_2_. TAP-treated dentine showed many well-spread cultured cells with spindle-shaped attachments to the dentine via lamellipodia and filopodia extending into the dentinal tubules. These conditions reflected the positive effect of TAP-treated dentine on the DPSCs since cellular attachment is known to be directly proportional to the viability and physiological conditions of the cells.^36^ The penetration of filopodia into the dentinal tubules is known to stabilise the DPSCs and provide appropriate adhesion.^37^ By contrast, most of the cells on the Ca(OH)_2_-treated dentine were still round-shaped and non-spreading cells. Only a few cells acquired well-spread spindle-shaped morphology in the later stages of culture. These findings revealed that Ca(OH)_2_ is less favourable in promoting the growth and attachment of DPSCs than TAP.^14^ This effect reflected the conditions that might happen clinically where this treatment might delay the complete root maturation of teeth treated with REPs.^6^^,^^38^ These findings were observed because we treated the dentine with medicaments to the maximum suggested 4 weeks. We believe that a previous study^17^ did not reveal this effect due to the shorter time of treatment applied. It is expected that the chemical action of Ca(OH)_2_ differs at different time points.

This study demonstrated the capability of TAP in promoting the growth and proliferation of DPSCs when cultured on a 4-week TAP-treated dentine in comparison with the those treated with Ca(OH)_2_. This study, however, was not without limitations. One limitation was the use of only premolars for the preparation of dentine; this does not represent dentine from other types of tooth dentine. The temperature of the dentine when treated with the medicaments was also not controlled to mimic body temperature. Moreover, different types of dental stem cell should be studied in future to investigate cell behaviour after treatment with these two medicaments. Furthermore, this study was an in vitro study; thus, the results only reflected the possible output of the procedure. Hence, an animal study should be conducted in future to confirm these findings.

## Conclusion

In the current study, TAP was proven to be a better medicament than Ca(OH)_2_ for 4 weeks of dentine treatment, as evidenced by the viability and attachment of DPSCs. This may represent promising evidence to support the current AAE disinfection protocol.

## Source of funding

This study was funded by a 10.13039/501100004713Universiti Sains Malaysia (USM), Malaysia Short Term Grant (reference: 304/PPSG/61313101).

## Conflict of interest

The authors have no conflicts of interest to declare.

## Ethical approval

Ethical approval was obtained on 24 December 2014 (reference: USM/JEPem/1406240) from the Human Research Ethics Committee of Universiti Sains Malaysia (JEPeM).

## Authors contributions

ATH and AA conceived and designed the study, and provided research materials. SR conducted research and collected and organised data. SR and AA analysed and interpreted data. SR, AA and ZM wrote the initial and final draft of the article and provided logistical support. All authors have critically reviewed and approved the final draft and are responsible for the content and similarity index of the manuscript.
